# Supplementary material for: The causal relationship between the human gut microbiota and pyogenic arthritis: a Mendelian randomization study
Source: Front Cell Infect Microbiol. 2024 Nov 26;14:1452480. doi: 10.3389/fcimb.2024.1452480 (PMC11629706; doi:10.3389/fcimb.2024.1452480)
Supplement: Supplementary file 1 [file DataSheet1.zip › mendelian test/ebi-a-GCST90027766.csv_forest.pdf]

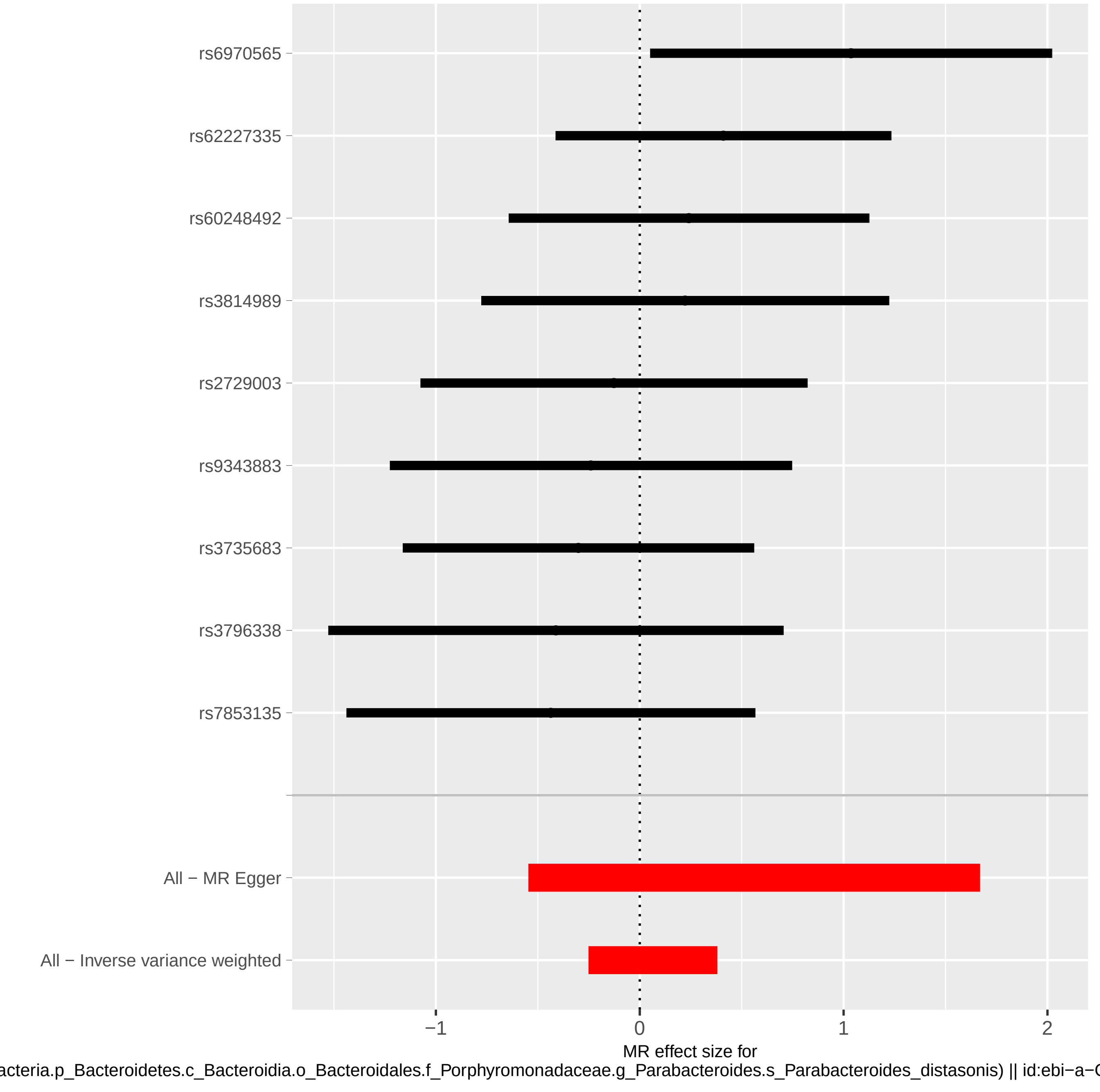
rs6970565
rs62227335
rs60248492
rs3814989
rs2729003
rs9343883
rs3735683
rs3796338
rs7853135
All − MR Egger
All − Inverse variance weighted
−1
0
1
2
MR effect size for
acteria.p_Bacteroidetes.c_Bacteroidia.o_Bacteroidales.f_Porphyromonadaceae.g_Parabacteroides.s_Parabacteroides_distasonis) || id:ebi−a−
